# Role of AMH in Prediction of Menopause

**DOI:** 10.3389/fendo.2021.733731

**Published:** 2021-09-14

**Authors:** Annelien C. de Kat, Frank J. M. Broekmans, Cornelis B. Lambalk

**Affiliations:** ^1^Department of Reproductive Medicine and Gynecology, University Medical Center Utrecht, Utrecht, Netherlands; ^2^Department of Reproductive Medicine, Amsterdam UMC, Vrije Universiteit, Amsterdam, Netherlands

**Keywords:** AMH, menopause, prediction, ovarian aging, reproduction

## Abstract

Anti-Müllerian Hormone (AMH) is produced by small antral follicles and has evolved over the past three decades as an assumed potential marker of the number of follicles in the human ovaries, also known as ovarian reserve. This quantitative measure, given the gradual decline over time and its non-replenishable feature, could be the dreamed marker for predicting the final exhaustion of ovarian storage: the post-menopause. This introductory chapter summarizes current knowledge with regard to the contribution of serum AMH measurements to predict age of normal menopause and critically discuss its potential in this regard. Furthermore, its predictive role in the context of menopause in association with several frequently occurring fertility disorders such as premature menopause, polycystic ovarian syndrome and endometriosis are discussed. Overall, while ovarian reserve markers including AMH are unmistakably related to age at menopause, they are insufficiently precise to inform on an individual’s journey of ovarian aging.

## Introduction

“Trying to predict the future is a loser’s game” (Ken Lui). Yet in medicine, prediction of future events is commonly used as a means of stratifying people into high or low risk and as an aid for treatment individualization. For every woman, the occurrence of menopause is a given, but the age at which she will enter menopause varies widely and is normally distributed between the ages of 40 to 60 (1). The onset of the menopausal transition indicates that the pool of resting and developing follicles in the ovaries, also known as ovarian reserve, is nearly depleted. Measuring the remaining ovarian reserve prior to this event could theoretically thus provide a risk estimation for the timing of the onset of menopause.

True ovarian reserve, i.e. the number of resting primordial follicles, can only be measured through histological tissue analysis. Available ovarian reserve tests therefore serve as a proxy for true ovarian reserve. The antral follicle count (AFC) measures the number of developing antral follicles by ultrasound, which is correlated to the size of the resting follicle pool (2). Levels of follicle-stimulating hormone (FSH) start to increase secondary to the age-related decline of estrogen-producing developing follicles. Rising FSH levels thus indicate a late stage of ovarian aging. Anti-Müllerian hormone (AMH) is produced by small developing, mostly antral, follicles. As these follicles are not yet responsive to FSH, AMH levels remain relatively stable throughout the menstrual cycle. In comparative studies, AMH was found to be the most favorable ovarian reserve marker for the prediction of age at menopause (3, 4).

## Age at Menopause Prediction

The majority of research efforts spent on the prediction of menopause stems from a desire to predict the duration of the reproductive lifespan. The rationale is usually that this enables a woman to gain information on the remaining time period she may have to become pregnant. Another objective could be to base a treatment strategy, such as ovarian surgery, on predicting the remaining years until the onset of menopause in a perimenopausal woman experiencing debilitating symptoms such as heavy menstrual bleeding or hot flashes. Whichever the objective, studies on the topic of menopause prediction require the assessment of desired predictors (such as age and AMH) at a baseline time point and a longitudinal follow-up period during which the event of interest (menopause) is recorded. Naturally, the study question dictates how much follow-up time is required. Statistical analysis of menopause prediction occurs through the development of prognostic models and a time-to-event analysis with a binary outcome (menopause or no menopause during follow-up), or the prediction of menopausal age as a continuous outcome.

Both statistical approaches have previously been utilized in menopause prediction studies. In all studies to date, AMH has proven to be a significant predictor for time to menopause or age at menopause (3, 5–12). The effect measure of AMH was presented in various ways; as an example, one unit decline in _log_AMH was associated with a 1.75-year earlier menopause (13), every unit increase in AMH was associated with decrease in the chance of becoming postmenopausal during follow-up illustrated by a hazard ratio (HR) of 0.092 [95% CI 0.025-0.340] (7)*;* women in the lowest AMH quartile had a 8.39 times higher risk of becoming postmenopausal during follow-up compared to women in the highest quartile (14). Prediction of individual age at menopause with the inclusion of AMH in the prediction model furthermore led to a similar distribution of predicted and observed ages at menopause (6, 9) and the observation that women in low age-specific AMH percentiles generally reached menopause at an earlier age than women with high age-specific AMH percentiles (7, 10).

## Methodological Pitfalls of Menopause Prediction

As can be expected, these results created much anticipation of the promise of AMH as a determinant of reproductive age. Indeed, a literature review of variables of menopause prediction concluded that AMH was the most promising predictor available (4). However, as the authors mention, this conclusion can be somewhat nuanced by a further exploration of the statistical analyses of these studies. Aside from statistical significance of an included predictor variable, the performance of prediction models should also be considered in their interpretation. In a pair of two women, the C-statistic provides a measure of how often the prediction model correctly identifies who will become postmenopausal during a set time period, based on their AMH levels (and other additional predictors in the model). Overall, the C-statistics in the aforementioned prediction studies were high (all above 80%), thus expressing a high degree of model discrimination. The addition of AMH to a similar model with age led to an overall improvement of the C-statistic, specifically from 84% to 92% (6); 87% to 90% (7), 85% to 92% (3) and 84% to 86% (10). While this improvement should be acknowledged, it is also clear that the added predictive effect of AMH to an already well-performing model is in fact modest. The C-statistic was not reported in two studies (5, 8).

Another factor to consider is the non-proportional predictive effect of AMH with age. With increasing age, the predictive capacity of AMH on top of age alone decreases (10). This may be interpreted in the sense that a regularly cycling woman at age 43, based on these characteristics alone, already has a far lower *a priori* chance of early menopause in comparison to a woman at age 30. Although this seems like an encouraging finding in menopause prediction for younger women, the downside is that the predictive *accuracy* of AMH is lower for women at younger ages. Thus, while AMH contributes more to a prediction model for a younger woman seeking to know whether she may enter menopause at a relatively early age, the predicted age range in which she will enter menopause is wider. A further methodological issue at play here is the relative lack of inclusion of women below the age of 30 years in the cohorts utilized in the aforementioned studies. The wide prediction intervals may therefore, at least in part, be influenced by insufficient statistical power.

One potential way to work around the limitation of menopause prediction with a single AMH measurement is to get an indication of the speed of the AMH decline. This may thus provide information on the kind of ovarian reserve decline trajectory a woman is on, with a swift decline theoretically leading to an earlier age at menopause. In a longitudinal analysis of 5 AMH measurements spanning a time period of 20 years, the speed of AMH decline was associated with AMH levels and found to vary with age (15). This suggests that a one-size-fits-all approach to AMH decline may be flawed. After the age of 25, knowledge of the prior 5-year AMH decline rate did not lead to improved C-statistics in comparison to the AMH level alone in the same cohort (11). In concordance with prior findings, AMH measurement at 20 and 25 years was associated with poorer C-statistics in comparison to AMH measurement at 30 years (62, 64 and 70% respectively). A more recent cohort study did find an improvement of C-statistics in menopause prediction with the addition of the AMH decline rate, calculated over a span of approximately 18 years: 70% to 78% (12). The latter finding has limited clinical applicability however, as this cannot be extrapolated to a short-term decline rate due to the large variation in AMH decline rate over time. It is unlikely that a woman seeking information on her future menopausal age, especially with the objective of family planning, will have the patience to wait 18 years for an improved prediction model.

Lastly, there are currently several AMH assays available for clinical use. These include the Gen II (Beckman Coulter), picoAMH (AnshLabs), AMH ELISA (AnshLabs), Elecsys (Roche) and Access (Beckman Coulter). Each assay has a different range of detection and sensitivity, which impedes the direct comparison and formulation of cut-off values of absolute serum AMH levels measured by different assays (16). Naturally, this means that if a well-performing model would be developed for AMH, its results would only directly apply to AMH measurements performed with the same assay, or require the application of a correction factor with an added risk of inaccuracy.

## Menopause Prediction in Clinical Subgroups

### Primary Ovarian Insufficiency

Primary ovarian insufficiency (POI) is defined by the permanent cessation of menses before the age of 40 years with substantially elevated levels of follicle-stimulating hormone (FSH). The situation when there is still a menstrual cycle but already elevated levels of FSH in the early follicular phase is called imminent ovarian insufficiency with the elevated FSH as the result of limited ovarian inhibin B feedback (17). Elevated levels of FSH are an irrefutable hormonal hallmark of reproductive aging. Unfortunately, longitudinal studies have shown that a markedly elevated FSH is a relatively late predictor of the menopausal transition, since increasing values only occur about 10 years before the menopause, which is probably also when infertility begins to prevail (18). Longitudinal studies have shown that inhibin B correlates with age only during a relatively short period before the menopause transition (19). A decrease in inhibin B seems the most important and earliest factor that plays a role in the elevation of early follicular phase FSH. Low or unmeasurable inhibin B levels theoretically could be used to indicate that the menopause is imminent. Unfortunately, its role as an early predictor is also limited. Thus, both elevated FSH and a declined Inhibin B seem appropriate predictors, but only just prior to the occurrence of menopause.

Current data indicate that measurement of AMH is a more accurate indicator of POI in many situations with diagnostic validity, and perhaps may facilitate more timely diagnosis although there are scarce data regarding prediction of POI far in advance. Within the prospective Nurses’ Health Study II cohort each 0.10 ng/ml decrease in AMH was associated with a 14% higher risk of early menopause supporting the potential utility of AMH as a clinical marker of early menopause in otherwise healthy women (20). However, due to inadequate precision, the ability of AMH to accurately predict the distant onset of POI seems as unreliable as for any age at menopause (21).

### Polycystic Ovary Syndrome

Polycystic ovary syndrome (PCOS) is a highly prevalent reproductive endocrine disorder characterized by a varying degree of hyperandrogenism, polycystic ovaries and anovulation which has some remarkable features with regard to reproductive ageing. Typically, women with previous anovulation almost all become ovulatory by the age of 40 (22). There are indications that PCOS is associated with a substantial delay of menopause by more than 4 years compared to regularly ovulating women. Prediction of age at (23) menopause using AMH in women with PCOS would correspond to an average extension of the reproductive lifespan by two years (24). With the assumption that higher AMH levels relate to later menopause, it is not unreasonable to interpret the substantially higher serum AMH levels in women with PCOS as a prelude to a later age at menopause. This then supports the notion that these higher AMH levels relate to a preexisting larger pool of follicles that exhausts later in life, aside from biochemical mechanisms that promote AMH secretion. Currently, substantially sized long-term follow-up studies relating previously measured AMH at a younger age to the actual age at menopause some years later in women with PCOS are not available.

### Endometriosis

Another condition of the reproductive system with concerns regarding fertility and age at menopause is endometriosis, and in particular ovarian endometriosis. Intuitively, an ovarian endometrioma may potentially affect ovarian reserve through its intrusion of ovarian tissue. To date only a few studies (25, 26) addressed this issue. According to Streuli et al. (25), endometriosis and ovarian endometriomas are not singularly related to lower AMH levels. In contrast, Uncu et al. (26) found that compared to controls, women with endometriomas did have lower AMH levels prior to surgery. Thus, there is still controversy here. A recent systematic review which compared AMH levels between women with uni- and bilateral endometrioma found no difference, which challenges the concept of damage to ovarian reserve by endometriomas. Surgical intervention is more consistently related to a sustained decline of ovarian reserve markers (27). Whether these lower AMH levels after the surgery predict earlier menopause, as may be expected, remains to be established. There are indications that this may be the case (28).

### Iatrogenic Ovarian Reserve Impairment

Women who have undergone treatment that may affect the pool of dormant primordial follicles represent a different category with regards to ovarian reserve measurement. In childhood cancer survivors, AMH levels appear to adequately reflect the ovarian reserve potential after gonadotoxic chemotherapy (23). Women who exhibited signs of preserved ovarian reserve after finalizing chemotherapy retained proportionally similar ovarian reserve status after 10 years of follow-up, which suggests that the decline of ovarian reserve may not be significantly altered (29). Indeed, in a longitudinal population study of childhood cancer survivors with detectable post-treatment AMH levels, the decline rate of AMH was very much comparable to that of a control population (30). It remains to be determined whether the prediction of age at menopause, albeit with a range spanning several years, may ultimately be feasible in this group of women facing reproductive decisions at a relatively early age. Of note, AMH levels are reduced in girls with newly diagnosed cancer even before the cancer treatment has started and it is suggested that possibly in relation to this impaired DNA repair mechanisms are involved that also seem in part to be involved in determination of age at menopause (31–33).

## Implications of Menopause Prediction

There is ample discussion as to whether and for whom menopause prediction can be applied in clinical practice. As previously highlighted, there are several groups of women who may benefit from a personalized estimate of the duration of their reproductive lifespan. For example, a young woman, considering whether she should opt for family or career first, could base her decision on potential biological restrictions. Indeed, younger women with a future desire for family planning were reportedly interested in testing for premature menopause (34). This willingness is capitalized on by companies offering ‘fertility’ tests, which often include an estimate of ovarian reserve. As discussed, while ovarian reserve markers are unmistakably related to age at menopause, they are insufficiently indicative of an individual’s journey of ovarian aging. The use of ovarian reserve markers for long-term predictions could therefore either lead to a false sense of security or unnecessary alarm. Similarly, a woman with a familial risk of POI may not benefit from an AMH measurement in the long-term, although the finding of a nearly depleted follicle pool may prompt her to take action in the short term. Still, the knowledge level of young women on their current and future fertility and the effects of increasing age is poor. Fertility tests may draw attention to these themes with the effect that women get informed on these topics and will be aware of the potential risks. Also, there is a need to understand and probe the way young women would handle results of fertility tests: if the early menopause (before age 45) hazard is 25% instead of 5%, based on the AMH test: what will young women do with such a result? This needs to come with information on the other player in this important field: average oocyte quality, which currently can only be captured by female age, but at the same time may highly vary within age categories. Understanding the interplay between follicle number and oocyte quality will help to understand that, in order to have a chance of at least 90% to naturally realize a two-child family, couples should start trying to conceive when the female partner is 27 years of age or younger (35). The role of AMH as a marker of ovarian aging in women with PCOS and endometriosis requires further elucidation, as there are multiple processes at play that may influence or be influenced by ovarian reserve in these heterogeneous disease entities.

## Author Contributions

All authors listed have made a substantial, direct, and intellectual contribution to the work, and approved it for publication.

## Conflict of Interest

The authors declare that the research was conducted in the absence of any commercial or financial relationships that could be construed as a potential conflict of interest.

## Publisher’s Note

All claims expressed in this article are solely those of the authors and do not necessarily represent those of their affiliated organizations, or those of the publisher, the editors and the reviewers. Any product that may be evaluated in this article, or claim that may be made by its manufacturer, is not guaranteed or endorsed by the publisher.
